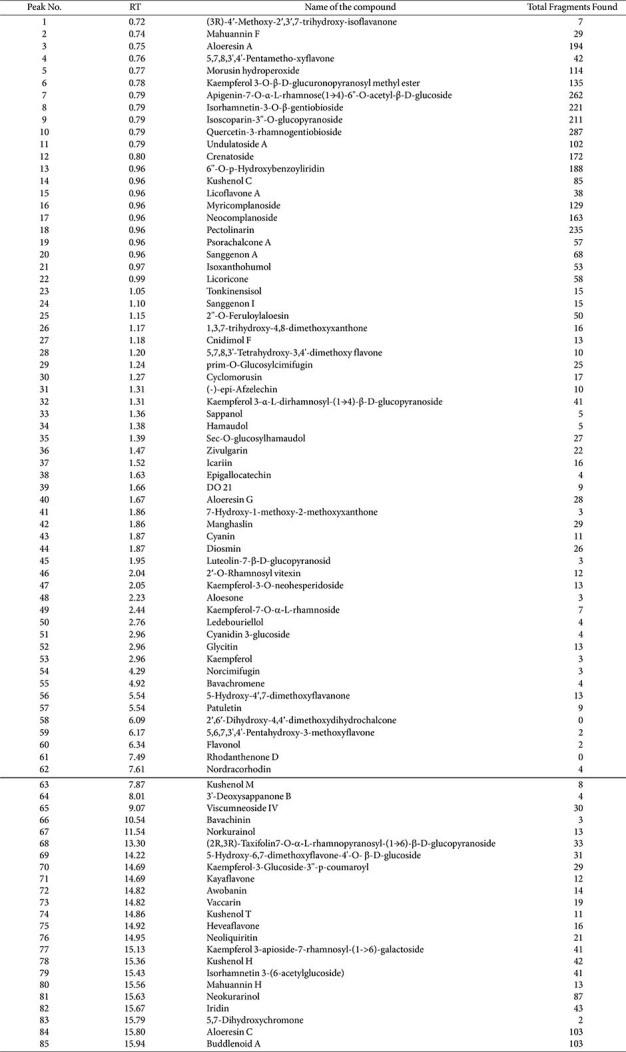# *In Vitro* Skin-Protective Effects of Sumyong Nature, a Kimchi- and Soybean-Derived Fusion-Fermented Microbial Supernatant, in Human Keratinocytes: Involvement of MAPK-Related Signaling

**DOI:** 10.4014/jmb.2605.05017

**Published:** 2026-07-07

**Authors:** Dilda Kaskadamova, Lei Huang, Nurinanda Prisky Qomaladewi, Natasha Christabella Sutopo, Ji Hye Yoon, Si Eun Yoon, Yerkyesh Khamit, Zhenyan Piao, Chae Kun Lee, Seung Yun Shin, Jae Youl Cho

**Affiliations:** 1 Department of Integrative Biotechnology, Sungkyunkwan University, Suwon 16419, Republic of Korea; 2 Department of Biocosmetics, Sungkyunkwan University, Suwon 16419, Republic of Korea; 3 Kunyoung ENC Co., Hwasung 93-54, Republic of Korea; 4 Pristineage, Seoul 348-9, Republic of Korea

**Keywords:** Sumyong Nature, Fermented microbial supernatant, UVB, Human keratinocytes, MMP-1, HAS-1, MAPK signaling

## Abstract

Sumyong Nature (SN) is a kimchi- and soybean-derived fusion-fermented microbial supernatant prepared from *Lactobacillus acidophilus*, *Saccharomyces cerevisiae*, *Weissella cibaria*, and *Bifidobacterium longum*. This study examined the *in vitro* effects of SN on UVB-induced damage and skin-related cellular responses in human keratinocytes. Cell viability assays, gene expression analysis, luciferase reporter assays, immunoblotting, scratch wound-healing assays, and LC-Q-TOF-MS analysis were conducted. SN showed no significant cytotoxicity in HaCaT or HEK293T cells at concentrations up to 75 μg/mL. In UVB-irradiated HaCaT cells, SN improved cell viability, suppressed MMP-1 expression, increased HAS-1 expression, and modulated UVB-responsive stress-related markers, including SOD-1, Nrf2, and HO-1. SN also promoted wound closure in keratinocytes and was associated with changes in NF-κB-, AP-1-, CREB-, and MAPK-related signaling responses under the tested conditions. LC-Q-TOF-MS analysis tentatively identified several candidate constituents, including pectolinarin and aloeresin-related compounds. Collectively, these findings suggest that SN exerts multiple beneficial *in vitro* effects in keratinocyte-based assays, warranting further mechanistic investigation and validation in advanced experimental models.

## Introduction

As the largest organ of the human body, the skin serves as an primary protective barrier against environmental stressors such as pathogens, UV radiation, and pollutants [1]. Its health and appearance are influenced by a wide range of intrinsic and extrinsic factors including genetics, lifestyle, and skincare habits. In recent years, increasing attention has been given to the importance of maintaining skin health not only for aesthetic purposes but also for preserving barrier function and preventing skin damage [2]. Structural integrity, proper moisturization, wrinkle-related changes, cellular defense against stress, wound repair, and barrier protection are all important aspects of healthy skin, especially in the context of premature aging and environmental damage [3].

Ultraviolet (UV) radiation from sunlight, particularly ultraviolet B (UVB), is one of the major external factors that contributes to premature skin photoaging [4]. UVB exposure induces cellular stress, DNA damage, and inflammatory responses in skin cells, ultimately contributing to visible and functional signs of skin deterioration [5, 6]. Because UVB-induced damage is closely linked to photoaging, identifying materials that can help protect keratinocytes from UVB-associated stress remains an important area of research. In this context, stress-responsive factors, including superoxide dismutase 1 (SOD-1), heme oxygenase-1 (HO-1), and the nuclear factor (erythroid-derived 2)-like 2 (Nrf2), are involved in cellular defense mechanisms [7, 8].

Wrinkle formation is one of the most visible signs of skin aging and is closely associated with the gradual decline of collagen and elastin fibers, a process that can be further accelerated by UVB exposure [9]. Matrix metalloproteinases (MMPs), particularly MMP-1, contribute to wrinkle formation by degrading collagen and other extracellular matrix components that maintain skin structure and elasticity. Another important factor in maintaining healthy skin is proper moisturization, which is essential for preserving barrier function and preventing transepidermal water loss (TEWL) [10, 11]. Hyaluronic acid (HA), produced by hyaluronan synthases (HAS), plays a central role in this process, and among these enzymes, HAS-1 participates in HA synthesis and may contribute to hydration- and barrier-related skin functions. [12-16]. In addition, efficient wound healing is important for restoring skin integrity and maintaining the protective function of the skin after injury or environmental stress [17]. Together, these factors make MMP-1, HAS-1, and wound-healing responses useful indicators for evaluating skin-related cellular effects *in vitro*. Because keratinocytes also participate in cutaneous immune responses, inflammatory cytokine stimulation provides another relevant context for evaluating the skin-related effects of SN. The interleukin-17 (IL-17) family, especially IL-17A, plays an important role in inflammatory and immune responses in the skin [18-22].

Crosstalk between IL-17A and cytokines such as IL-4, IL-13, and IL-31, as well as interactions with sensory-associated ion channels such as TRPV1, has also been implicated in inflammatory skin conditions [23-26]. Although the present study does not aim to establish a therapeutic model for these diseases, these pathways provide relevant background for understanding selected inflammation- and sensory-related markers evaluated in keratinocytes.

*Lactobacillus acidophilus*, *Saccharomyces cerevisiae*, *Bifidobacterium longum* and *Weissella cibaria* are microorganisms associated with fermented foods and have been investigated in probiotic, postbiotic, and functional food contexts. Fermented foods and microbial products have long been used in various traditional dietary and medicinal contexts, including traditional Korean medicine, Ayurveda, and traditional Chinese medicine [27]. In recent years, probiotics, postbiotics, and fermentation-derived microbial materials have attracted growing interest as potential ingredients for skin-related applications [28]. Among the microorganisms mentioned above, *L. acidophilus*, *S. cerevisiae*, *W. cibaria*, and *B. longum* have each drawn attention for possible roles in skin health. Previous studies have suggested that *L. acidophilus*-derived preparations have been reported to affect wrinkle-related markers, including matrix metalloproteinases [29]. *S. cerevisiae* may show anti-inflammatory or wound-healing–related effects [30,31]. *W. cibaria* may improve atopic dermatitis-like symptoms [32], and *B. longum* lysate may strengthen the skin barrier and reduce skin sensitivity [33]. Nevertheless, the skin-related effects of fusion-fermented microbial supernatants prepared from these microorganisms remain insufficiently characterized, particularly in keratinocyte-based *in vitro* systems.

In this study, we investigate the *in vitro* effects of Sumyong Nature (SN), a kimchi- and soybean-derived fusion-fermented microbial supernatant prepared from *L. acidophilus*, *S. cerevisiae*, *W. cibaria*, and *B. longum*, on UVB-induced and skin-related cellular responses in human keratinocytes. We focused on UVB-induced cytotoxicity, MMP expression, HAS-1 expression, wound-healing–related activity, selected inflammatory markers, chemical profiling, and associated signaling responses.

## Materials and Methods

### Materials

Immortalized human keratinocytes (HaCaT cells) and human embryonic kidney 293T cells (HEK293T) cells were purchased from the American Type Culture Collection (USA). Various reagents and chemicals were sourced from multiple suppliers: fetal bovine serum (FBS), penicillin-streptomycin, Dulbecco’s modified Eagle’s medium (DMEM), phosphate-buffered saline (PBS), and trypsin were obtained from Hyclone (USA). 3-(4,5-dimethylthiazol-2-yl)-2,5-diphenyl tetrazolium bromide (MTT), TRIzol reagent, polyethyleneimine (PEI), bovine serum albumin (BSA), quercetin, gallic acid, aluminum chloride, and ascorbic acid were purchased from Sigma Aldrich Chemical Co. (USA). MTT stopping solution was prepared by combining 10% sodium dodecyl sulfate with hydrochloric acid (HCl). The cDNA synthesis kit was obtained from Thermo Fisher Scientific (USA). Forward and reverse primers used in the qPCR and RT-PCR experiments were synthesized by Macrogen (Republic of Korea), and the PCR premix was obtained from Bio-D Inc. (Republic of Korea). The luciferase assay system kit was purchased from Promega (USA). Polyvinylidene difluoride (PVDF) membranes and enhanced chemiluminescence (ECL) reagent were acquired from Bio-Rad (USA). Specific antibodies for the total and phosphorylated forms of c-Fos, c-Jun, ERK, p38, JNK, IKKα, IκBα, p50, and p65 were obtained from Cell Signaling Technology (USA), and SOD-1, Nrf2, HO-1 and β-actin were purchased from Santa Cruz Biotechnology (USA).

### Preparation of the Culture Medium for SN Fermentation

First, 6% by weight of leaf extracts from *Ficus carica*, *Prunus persica*, *Prunus mume*, *Castanea crenata*, *Phyllostachys bambunosides*, and *Pinus densiflora* were combined with distilled water. Subsequently, 3% by weight of okara and 1% by weight of molasses were added to the mixture to create the culture medium.

### Preparation of the Proprietary Fusion-Fermented Microbial Supernatant, Sumyong Nature (SN)

SN was used as a proprietary kimchi- and soybean-derived fusion-fermented microbial supernatant prepared from *L. acidophilus*, *S. cerevisiae*, *W. cibaria*, and *B. longum*. The resulting fermented supernatant was freeze-dried to obtain a powder, designated as Sumyong Nature.

For fermentation, *L. acidophilus* was first cultured in a medium containing molasses (10 g/L) at 37°C for 10 days under anaerobic condition. Thereafter, *B. longum* and *W. cibaria* were added and cultured for an additional 10 days at 37°C in the presence of molasses (100 g/L). An additional fermentation step was then carried out at 35°C under anaerobic conditions for 10 days, followed by a second fermentation step at 45°C for another 10 days under oxygen-supplemented conditions. The resulting culture medium was filtered, and the liquid fraction was lyophilized under vacuum at -85°C and 5 mTorr.

### Liquid Chromatography-Q-Tof-Mass Spectrometry

LC-Q-Tof-MS analysis was performed to profile the chemical constituents of SN. Xevo G2-XS Q-TOF-LC/MS (Waters, USA) was used for the analysis as previously described. Data acquisition was performed using Waters MassLynx Software 4.2 and UNIFI Portal Software (Waters).

### Cell Lines and Cell Culture Conditions

HaCaT and HEK293T cells were cultured in DMEM supplemented with 10% (v/v) heat-inactivated fetal bovine serum (FBS) and 5% (v/v) heat-inactivated FBS, respectively, along with 1% (v/v) penicillin-streptomycin antibiotics. All cell lines were cultured and incubated in a 5% CO_2_ incubator at 37ºC.

### Cell Viability Assay

A conventional MTT assay was performed to determine the cytotoxicity of SN. The HaCaT and HEK293T cells were seeded in 96-well plates at a density of 5 × 10^5^ cells/mL and 2 × 10^5^ cells/mL, respectively, and incubated overnight. The cells were then treated with SN at 25, 50, and 75 μg/mL. After 24 h, 100 μL of the culture medium was removed, and MTT solution was added to each well. After incubation for 3 h, 100 μL of MTT stopping solution was added to each well. The absorbance was measured the following day at 570 nm using a microplate reader.

### Cell Morphology

HaCaT cells were seeded in six-well plates at a density of 2 × 10^5^ cells/mL and incubated overnight. The cells were pretreated with SN (0-75 μg/mL) for 30 min, exposed to UVB irradiation (30 mJ/cm^2^), and then re-treated with SN. After incubation for 12 h, cell morphology was observed and images were captured using an epifluorescence microscope (Olympus, Japan).

### UVB Treatment

HaCaT cells were seeded in six-well plates at a density of 3 × 10^5^ cells/well and incubated overnight. The cells were pretreated with SN for 30 min before UVB exposure. After washing with PBS, the cells were exposed to UVB irradiation at 30 mJ/cm^2^. After irradiation, PBS was removed, and the cells were re-treated with SN and incubated at 37°C in a 5% CO_2_ incubator.

### Melanin Secretion and Content Analysis

Melanin secretion and content assays were performed following the modified method described previously [34]. B16F10 cells (2.5 × 10^5^ cells/mL) were seeded in 12-well plates and incubated for 20 h. The cells were treated with α-MSH in the presence or absence of SN or arbutin. Melanin secretion was determined by measuring the absorbance of the cell culture medium at 475 nm. For the melanin content assay, B16F10 cells were harvested and analyzed as previously described [34].

### Plasmid Transfection (NF-κB/AP-1/CREB) and Luciferase Reporter Gene Activity Assay

HEK293T cells were seeded at a density of 1 × 10^5^ cells/mL and transfected with β-galactosidase and *NF-κB*-Luc, *AP-1*-Luc, or *CREB*-Luc reporter plasmids (0.8 μg/mL) using PEI. After incubating for 24 h, cells were treated with different concentrations of SN and then further incubated for another 24 h. The culture medium was then removed, and 300 μL of luciferase lysis buffer was added to each well. The lysates were stored at -70°C overnight prior to analysis. Luciferase activity was measured as previously described, and luminescence readings were normalized to β-galactosidase activity.

### RNA Extraction and Semi-Quantitative Reverse-Transcription Polymerase Chain Reaction (RT-PCR)

HaCaT cells were plated in six-well plates at a density of 5 × 10^5^ cells/mL and incubated overnight. The next day, the cells were treated with SN and incubated for 24 h. The cells were then harvested with cold PBS and centrifuged at 5000 rpm for 3 min. Total RNA was extracted using TRIzol reagent, and its concentration measured using a spectrophotometer. cDNA was synthesized using a cDNA synthesis kit according to the manufacturer’s instructions. Semi-quantitative RT-PCR was then performed under the following conditions; initial denaturation at 98°C for 5 min, denaturation at 98°C for 15 s, annealing at 56°C–61°C for 15 s, extension at 72°C for 1 min, and a final extension at 72°C for 5 min. The sequences of all the primer sets used in this study are listed in Table 1.

### Total Cell Lysate Preparation

HaCaT cells were seeded in six-well plates at a density of 3 × 10^5^ cells/mL and incubated overnight. The next day, the cells were treated with different concentrations of SN for 24 h. After treatment, the cells were washed with cold PBS. The harvested cells were then centrifuged at 12,000 rpm for 5 min at 4ºC. The supernatant was removed, and cell pellets were lysed on ice for 15 min using cell lysis buffer containing 20 mM Tris-HCl (pH 7.4), 2 mM EDTA, 2 mM ethylene glycol tetraacetic acid (EGTA), 1 mM dithiothreitol, 50 mM β-glycerol phospshate, 0.1 mM sodium vanadate, 1.6 mM pervanadate, 1% Triton X-100, 10% glycerol, 10 μg/mL aprotinin, 10 μg/mL pepstatin, 1 mM benzamidine, and 2 mM phenylmethylsulfonyl fluoride. The lysates were then collected and stored at -70°C until use.

### Immunoblotting Analysis

The lysates were centrifuged at 12,000 rpm for 1 min at 4°C to remove cell debris, and protein concentrations were determined using the Bradford protein assay (BIO-RAD, USA). Equal amounts of protein (20 μg per lane) were separated by Tris-glycine SDS gels and then transferred onto PVDF membranes. The membranes were blocked with 3% BSA for 1 h at room temperature, washed three times with Tris-buffered saline containing 0.1% Tween-20 (TBST), and then incubated with primary antibodies overnight at 4°C. After washing with TBST three times for 10 min each, the membranes were incubated with secondary antibodies for 4 h at room temperature. After an additional round of washing with TBST, immunoreactive bands were visualized using EzWestLumi plus (ATTO Corporation, Taito-ku, Japan).

### Wound Healing Assay

HaCaT cells were seeded in six-well plates at a density of 4 × 10^4^ cells/mL and incubated at 37°C in a 5% CO_2_ incubator for 24 h until the cells reached complete confluence. Scratch wounds of 0.5–0.9 mm in width were created using a sterile pipette tip (Ø = 0.1 mm). After washing with PBS to remove detached cells and debris, the cells were treated with SN at concentrations of 25, 50, and 75 μg/mL. Images of each scratched area were captured at 0, 6, and 24 h using a microscope.

### IL-17A Stimulation in HaCaT Cells

HaCaT cells were plated in six-well plates at a density of 5 × 10^5^ cells/mL and incubated at 37°C in a 5% CO_2_ incubator for 24 h. The next day, the cells were treated with SN at concentrations of 25, 50, and 75 μg/mL for 30 min. The cells were then treated with IL-17A for an additional 2 h. After treatment, the cells were harvested, and mRNA was extracted as described above.

### Statistical Analysis

All data are presented as means ± standard deviations of at least two independent experiments. The graphs were drawn in SigmaPlot (Systat Software, USA). For statistical analysis, the data were compared between experimental groups using Student’s *t*-test. *P*-values < 0.05 were considered statistically significant.

## Results

### Effects of SN on Cell Viability in HaCaT and HEK293T Cells

**Fig. 1:**
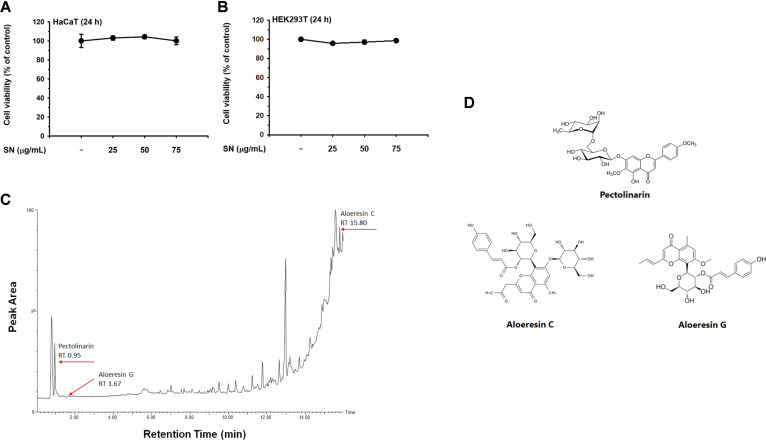
Effect of SN on cell viability and chemical profiling. (**A**) HaCaT cells were treated with different concentrations of SN (25–75 μg/mL) for 24 h, and cell viability was measured using the MTT assay. (**B**) The cell viability of HEK293T cells treated with SN (25–75 μg/mL) for 24 h was measured using the MTT assay. (**C**) LC-Q-TOF-MS chromatographic profile of SN. (**D**) Chemical structure of representative candidate constituents tentatively identified in SN.

To assess the cytotoxicity of SN, cell viability was evaluated in HaCaT and HEK293T cells using the MTT assay [35]. As shown in Fig. 1A and 1B, SN did not significantly reduce cell viability in either HaCaT or HEK293T cells at concentrations up to 75 μg/mL, indicating no apparent cytotoxicity under the tested conditions.

### Chemical Profiling of SN by LC-Q-TOF-MS

To explore the chemical constituents of SN, LC-Q-TOF-MS analysis was performed. As shown in Fig. 1C, several chromatographic peaks were detected, and candidate compounds, including pectolinarin, aloeresin G, and aloeresin C, were tentatively identified. The chemical structures of representative candidate constituents are shown in Fig. 1D, and the corresponding profiling data are summarized in Table 2. Previous studies have suggested that some of these compounds may have skin-related bioactivities, including skin-protective or moisturizing potential [36]. However, the present study did not determine the direct contribution of individual compounds to the observed effects of SN. Therefore, these constituents should be regarded as candidate bioactive components of SN, and their precise roles will require further validation.

### Skin-Protective Effects of SN against UVB-Induced Damage

**Fig. 2:**
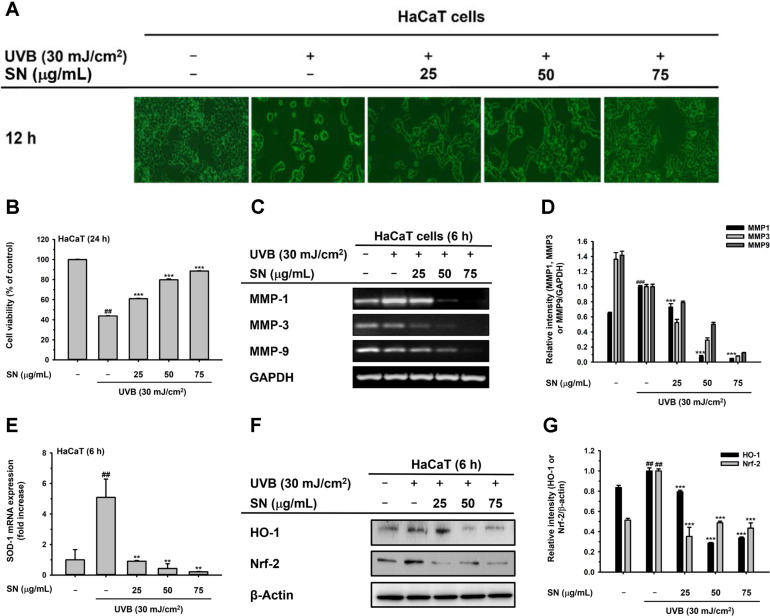
Effects of SN on UVB-induced cellular responses in HaCaT cells. (**A**) Morphological changes in HaCaT cells treated with SN (25, 50, 75 μg/mL) under UVB irradiation were examined after 12 h by microscopy. (**B**) Cell viability of HaCaT cells exposed to UVB (30 mJ/cm^2^) and treated with SN was measured using the MTT assay after 24 h. (**C and D**) Expression levels of MMP1, MMP3, and MMP9 in HaCaT cells exposed to UVB (30 mJ/cm^2^) and treated with SN were assessed by RT-PCR after 6 h. (**E**) The relative band intensities of MMP-1, MMP-3, and MMP-9 were quantified using ImageJ software. (**E**) Expression of SOD-1 in HaCaT cells exposed to UVB (30 mJ/cm^2^) and treated with SN for 6 h was assessed by real-time PCR. (**F and G**) Protein expression levels of HO-1 and Nrf2 in HaCaT cells exposed to UVB irradiation (30 mJ/cm^2^) and treated with SN for 6 h were analyzed by immunoblotting. (**G**) The relative band intensities of HO-1 and Nrf-2 were quantified using ImageJ software. Data are presented as the mean ± standard deviation. p < 0.05, ** p < 0.01 and *** p < 0.001 compared with the UVB-treated group. ^#^ *p* < 0.05, ^##^ *p* < 0.01 and ^###^ *p* < 0.001 compared with the untreated normal group.

To further examine the protective effects of SN against UVB-induced damage, morphological changes and cell viability were evaluated in HaCaT cells after UVB exposure. As shown in Fig. 2A, UVB irradiation markedly altered cell morphology, whereas SN treatment attenuated UVB-induced morphological alterations in HaCaT cells after 12 h. In addition, UVB exposure significantly reduced cell viability, while treatment with SN at 25, 50, and 75 μg/mL improved cell viability in a concentration-dependent manner (Fig. 2B). These findings suggest that SN exerts protective effects against UVB-induced cellular damage in HaCaT cells under the tested conditions.

### Effects of SN on UVB-Induced MMP Expression in HaCaT Cells

Wrinkle formation is associated with multiple factors, including aging, reduced skin elasticity, collagen loss, and chronic sun exposure. In particular, repeated UV irradiation accelerates wrinkle formation by damaging collagen and elastin fibers in the skin, thereby promoting premature aging [37]. To investigate whether SN exerts anti-wrinkle–related effects in human keratinocytes, we examined the expression of wrinkle-associated genes, including MMP-1 (collagenase), MMP-3 (stromelysin), and MMP-9 (gelatinase), which are involved in extracellular matrix remodeling and degradation in the skin [34]. Because UV irradiation is known to increase MMP expression in skin cells, HaCaT cells were pretreated with SN for 30 min, exposed to UVB (30 mJ/cm^2^), and then further incubated with SN for 6 h. As shown in Fig. 2C and 2D, SN reduced the UVB-induced upregulation of MMP-1, MMP-3, and MMP-9, with MMP-1 showing a clear reduction compared with the UVB-treated group without SN. These findings suggest that SN may exert anti-wrinkle–related effects under the tested conditions.

### Effects of SN on UVB-Responsive Stress-Related Markers

To further investigate whether SN modulates UVB-responsive stress-related markers, the expression of SOD-1, Nrf2, and HO-1 was examined under UVB-exposed conditions. As shown in Fig. 2E, UVB irradiation increased SOD-1 expression, whereas SN treatment reduced this UVB-induced upregulation. Similarly, SN decreased the elevated protein levels of Nrf2 and HO-1 under UVB exposure (Fig. 2F and 2G). These findings indicate that SN modulates UVB-responsive stress-related markers under the tested conditions.

### Effect of SN on IL-17A-Stimulated Cytokine Responses

**Fig. 3:**
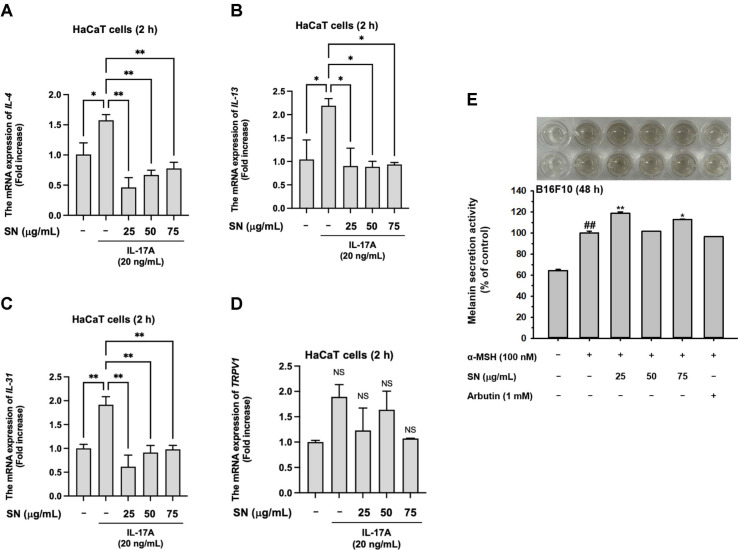
Effect of SN on selected IL-17A-responsive markers in HaCaT cells and α-MSH-induced melanogenic response in B16F10 cells. (**A-D**) mRNA expression levels of IL-4, IL-13, IL-31, and TRPV1 were measured in HaCaT cells after treatment with SN and IL-17A for 2 h. (**E**) Melanin secretion in B16F10 cells treated with α-MSH, SN, or arbutin was measured by absorbance at 475 nm. All results are presented as mean ± standard deviation. For (A–D), * *p* < 0.05 and ** *p* < 0.01 compared with the IL-17A-treated group, and ^#^ *p* < 0.05 and ^##^ *p* < 0.01 compared with the untreated normal group. For (**E**), * *p* < 0.05 and ** *p* < 0.01 compared with the a-MSH-treated group, and ^#^ *p* < 0.05 and ^##^ *p* < 0.01 compared with the untreated group.

We next examined whether SN modulates selected cytokine responses under IL-17A stimulation in keratinocytes. As shown in Fig. 3A and 3B, SN significantly reduced the IL-17A-induced upregulation of IL-4 and IL-13 compared with the group treated with IL-17A alone. In contrast, the responses of IL-31 and TRPV1 showed more variable patterns across the tested concentrations (Fig. 3C and 3D). Although changes in their expression were observed, these responses were less consistent than those observed for IL-4 and IL-13. Taken together, these results suggest that SN modulates selected IL-17A-responsive markers in keratinocytes.

### Effect of SN on α-MSH-Induced Melanogenic Response in B16F10 Cells

We further examined whether SN affects melanogenic responses in B16F10 cells. Because melanin production is stimulated by α-MSH, B16F10 cells were treated with α-MSH in the presence or absence of SN, and arbutin was used as a positive control [38]. As shown in Fig. 3E, the α-MSH-treated group exhibited increased melanin secretion, whereas arbutin reduced melanin secretion. In contrast, SN-treated groups did not show a clear concentration-dependent decrease in melanin secretion under the tested conditions. These results suggest that SN does not exert a marked inhibitory effect on α-MSH-induced melanogenesis in B16F10 cells.

### Moisturizing-Related Effects of SN in HaCaT Cells

**Fig. 4:**
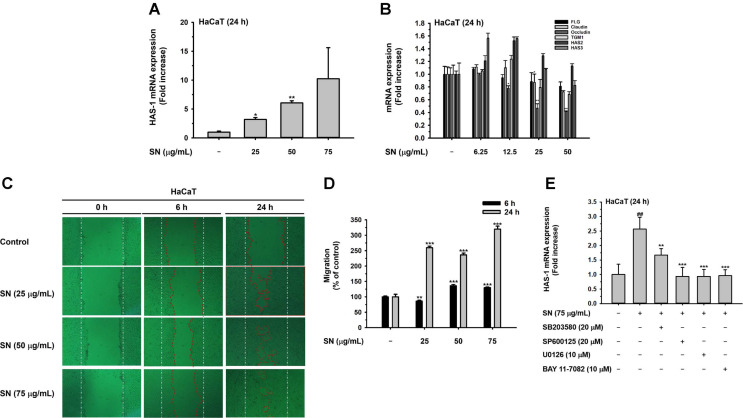
Moisturizing-related and wound-healing–related effects of SN in HaCaT cells. (**A**) mRNA expression of HAS-1 in HaCaT cells treated with SN was determined by real-time PCR. (**B**) Expression levels of FLG, Claudin, Occludin, TGM1, HAS-2, and HAS-3 in HaCaT cells treated with SN were analyzed by real-time PCR. (**C**) Representative images of the scratch wound assay in HaCaT cells treated with different concentrations of SN for 6 h and 24 h. (**D**) Relative wound closure in SN-treated groups was quantified using ImageJ analysis. (**E**) HAS-1 expression in HaCaT cells treated with SN in the presence or absence of SB203580, SP600125, U0126, and BAY 11-7082 was measured by real-time PCR. All results are presented as mean ± standard deviation. * *p* < 0.05, ** *p* < 0.01 and *** *p* < 0.001 compared with the control group. ^#^ *p* < 0.05, ^##^ *p* < 0.01 and ^###^ *p* < 0.001 compared with the untreated normal group.

Maintaining proper hydration is important for skin elasticity, barrier function, and overall skin condition. To examine the moisturizing-related effects of SN, the expression of hydration-associated genes was evaluated in HaCaT cells by real-time PCR. As shown in Fig. 4A, SN increased the expression of HAS-1 in a concentration-dependent manner at 25, 50, and 75 μg/mL. In contrast, other hydration- and barrier-related markers, including FLG, claudin, occludin, TGM-1, HAS-2, and HAS-3, did not show a clear concentration-dependent increase under the tested conditions (Fig. 4B). To further examine whether signaling pathways may be associated with SN-induced HAS-1 upregulation, cells were co-treated with SN and inhibitors of p38 MAPK (SB203580), JNK (SP600125), MAPK/ERK kinase (U0126), or NF-κB signaling (BAY 11-7082). As shown in Fig. 4E, HAS-1 expression was increased in the group treated with 75 μg/mL SN alone, whereas this increase was attenuated in the presence of each inhibitor. These findings suggest that SN-induced HAS-1 upregulation is associated with multiple signaling pathways under the tested conditions.

### Wound-Healing–Related Effects of SN in Keratinocytes

To evaluate whether SN affects wound-healing–related responses, a scratch wound assay was performed in HaCaT cells treated with SN. As shown in Fig. 4C and 4D, wound closure was enhanced in the SN-treated groups compared with the untreated group at both 6 h and 24 h. In particular, treatment with 50 and 75 μg/mL SN showed a more pronounced increase in wound closure over time. These results suggest that SN promotes wound-healing–related activity in keratinocytes under the tested conditions.

### Effects of SN on NF-κB-Related Signaling Responses

**Fig. 5:**
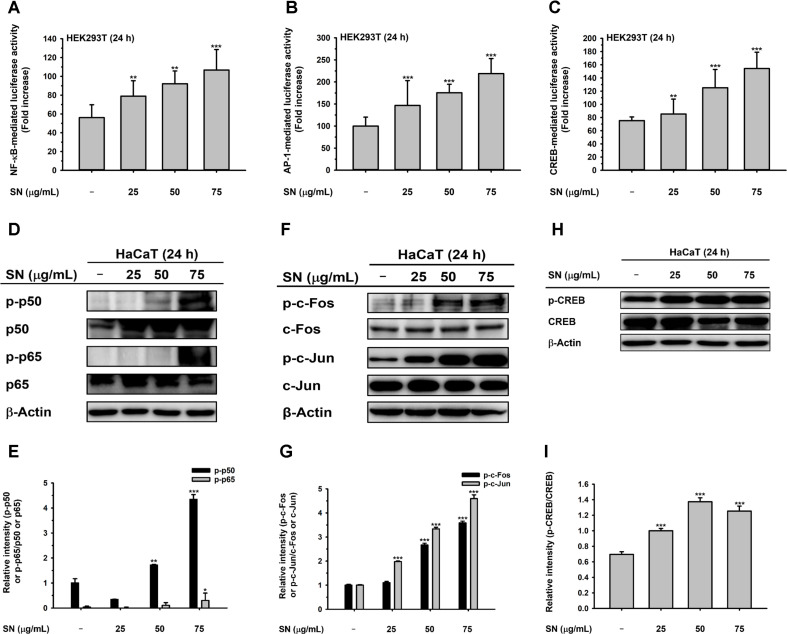
Effects of SN on *NF-κB*-, *AP-1*-, and *CREB*-related signaling responses. (**A-C**) Luciferase reporter assays were performed in HEK293T cells transfected with NF-κB-Luc, AP-1-Luc or CREB-Luc together with β-galactosidase as an internal control. Cells were treated with SN for 24 h, and luciferase activity was measured using a luminometer. (**D**) Immunoblotting analysis of NF-κB-related signaling proteins in HaCaT cells treated with SN for 24 h. (**E**) Relative band intensities of p-p50/p50 and p-p65/p65 were quantified using ImageJ analysis. (**F**) Immunoblotting analysis of AP-1-related signaling proteins in HaCaT cells treated with SN for 24 h. (**G**) Relative band intensities of p-c-Fos/c-Fos and p-c-Jun/c-Jun were quantified using ImageJ analysis. (**H**) Immunoblotting analysis of CREB-related signaling protein in HaCaT cells treated with SN for 24 h. (**I**) Relative band intensities of p-CREB/CREB were quantified using ImageJ analysis. All results are presented as mean ± standard deviation. * *p* < 0.05, ** *p* < 0.01 and *** *p* < 0.001 compared with the control group. ^#^ *p* < 0.05, ^##^ *p* < 0.01 and ^###^ *p* < 0.001 compared with the untreated group.

**Fig. 6:**
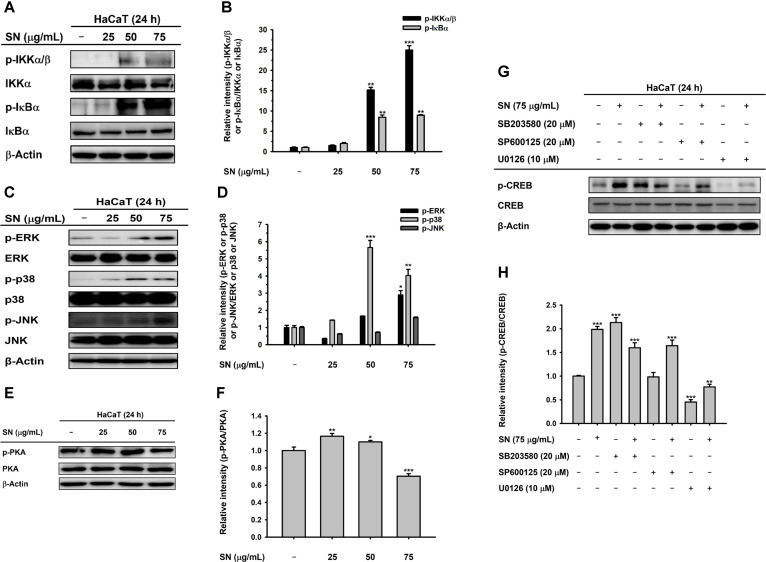
Effects of SN on *NF-κB-*, *AP-1-*, and *CREB*-related signaling proteins in HaCaT cells. (**A**) Immunoblotting analysis of NF-κB-related upstream signaling proteins in HaCaT cells treated with SN for 24 h. (**B**) Relative band intensities of p-IKKα/β/IKKα and p-IκBα/IκBα were quantified using ImageJ analysis. (**C**) Immunoblotting analysis of MAPK-related signaling proteins in HaCaT cells treated with SN for 24 h. (**D**) Relative band intensities of p-ERK/ERK, p-p38/p38, and p-JNK/ JNK were quantified using ImageJ analysis. (**E**) Immunoblotting analysis of PKA-related signaling proteins in HaCaT cells treated with SN for 24 h. (**F**) Relative band intensities of p-PKA/PKA were quantified using ImageJ analysis. (**G**) HaCaT cells were pretreated with SB203580, SP600125, or U0126 for 30 min, followed by treatment with SN (75 μg/mL) for 24 h. p-CREB levels were analyzed by immunoblotting. (**H**) Relative band intensities of p-CREB/CREB were quantified using ImageJ analysis. All results are presented as mean ± standard deviation. * *p* < 0.05, ** *p* < 0.01 and *** *p* < 0.001 compared with the control group. ^#^ *p* < 0.05, ^##^ *p* < 0.01 and ^###^ *p* < 0.001 compared with the untreated group.

Because SN increased HAS-1 expression and the SN-induced upregulation of HAS-1 was attenuated in the presence of signaling inhibitors, we further examined whether SN modulates NF-κB-related signaling responses under the tested conditions. First, an NF-κB luciferase reporter assay was performed. As shown in Fig. 5A, treatment with 25, 50, and 75 μg/mL of SN increased NF-κB luciferase activity and phosphorylation of NF-κB-related signaling proteins under non-inflammatory conditions. We next examined NF-κB-associated signaling proteins by immunoblotting. As shown in Fig. 5D and 5E, treatment with SN at concentrations of 25, 50, and 75 μg/mL increased the phosphorylation levels of p50 and p65. In addition, upstream signaling molecules related to the NF-κB pathway were examined, and SN increased the phosphorylation levels of IKKα/β and IκBα, whereas the total forms remained largely unchanged (Fig. 6A). These findings indicate that SN modulates NF-κB-related signaling responses under the experimental conditions used in this study.

### Effects of SN on AP-1/MAPK-Related Signaling Responses under Non-UVB Conditions

MAPK/AP-1 signaling has been implicated in various skin-related cellular responses [39-41]. To examine whether SN modulates AP-1/MAPK-related signaling under non-UVB conditions, we first performed an AP-1 luciferase reporter assay. As shown in Fig. 5B, treatment with 25, 50, and 75 μg/mL of SN increased AP-1 luciferase activity in HEK293T cells compared with the untreated group. We next examined AP-1-associated signaling proteins in HaCaT cells and found that treatment with 25, 50, and 75 μg/mL of SN increased the phosphorylation levels of c-Fos and c-Jun (Fig. 5F and 5G). In addition, because SN-induced HAS-1 expression was attenuated by SB203580, SP600125, and U0126, inhibitors of p38 MAPK, JNK, and MAPK/ERK kinase, respectively, we further examined MAPK-related signaling proteins. As shown in Fig. 6C and 6D, treatment with 25, 50, and 75 μg/mL of SN increased the phosphorylation levels of ERK, p38, and JNK, whereas the total forms remained largely unchanged. These findings indicate that SN modulates AP-1/MAPK-related signaling responses under the tested conditions.

### Effects of SN on CREB-Related Signaling Responses

To examine whether SN modulates CREB-related signaling under the tested conditions, we performed a CREB luciferase reporter assay. As shown in Fig. 5C, treatment with 25, 50, and 75 μg/mL of SN increased CREB luciferase activity compared with the untreated group. We next examined CREB phosphorylation by immunoblotting and found that treatment with 25, 50, and 75 μg/mL of SN increased the level of p-CREB, whereas the total CREB level remained largely unchanged (Fig. 5H and 5I). Because previous studies have suggested that CREB activity can be influenced by MAPK-related signaling [13, 42, 43], we further examined upstream kinase responses. As shown in Fig. 6E and 6F, SN did not produce a clear concentration-dependent increase in p-PKA. We therefore further examined CREB-related signaling in the presence of MAPK inhibitors. As shown in Fig. 6G and 6H, p-CREB levels remained elevated in cells co-treated with SN and SP600125 or U0126. Taken together, these findings suggest that SN modulates CREB-related signaling responses under the tested conditions.

## Discussion

The skin is a multifunctional organ that plays essential roles in protecting the body from environmental stressors and maintaining overall physiological homeostasis [1]. Among various environmental insults, UVB irradiation is widely recognized as a major contributor to skin aging, leading to wrinkles, loss of elasticity, and impaired skin barrier function [4]. UVB exposure also disrupts skin moisture balance and triggers cellular stress responses that contribute to skin damage [44, 45]. In this context, postbiotic or fermentation-derived materials have attracted increasing attention for their potential skin-related applications. In the present study, we investigated the effects of SN, a fermentation-derived microbial supernatant prepared from *L. acidophilus*, *S. cerevisiae*, *W. cibaria*, and *B. longum* in keratinocyte-based models of UVB-induced damage and skin-related cellular responses. The present findings showed that SN did not exhibit significant cytotoxicity in HaCaT or HEK293T cells at concentrations up to 75 μg/mL. In addition, SN improved cell viability in UVB-irradiated HaCaT cells in a concentration-dependent manner, suggesting that SN may protect keratinocytes from UVB-induced cellular damage *in vitro*. Along with this protective effect on cell viability, SN reduced the UVB-induced increase in SOD-1 mRNA expression and decreased the elevated protein levels of Nrf2 and HO-1. Interestingly, although antioxidant responses are commonly associated with activation of the Nrf2 pathway, the present pattern does not support a simple interpretation as direct activation of canonical antioxidant signaling. Instead, these findings may reflect attenuation of the overall cellular stress response triggered by UVB exposure, thereby reducing the compensatory induction of these stress-responsive markers. Therefore, the current data are more consistent with modulation of UVB-induced cellular stress responses than with direct activation of the Nrf2/HO-1 antioxidant pathway. Because intracellular ROS levels were not directly measured in the present study, it remains unclear whether SN acted upstream by reducing ROS levels or through another regulatory mechanism. This should be considered a limitation of the present study, and the precise upstream mechanism responsible for the reduction of UVB-induced Nrf2, HO-1, and SOD-1 requires further investigation.

Wrinkle formation is one of the most visible features of skin aging and is closely associated with extracellular matrix degradation [46]. In particular, MMP-1 plays a key role in collagen breakdown and wrinkle formation under stress conditions such as UVB exposure [9]. In the present study, SN suppressed UVB-induced MMP-1 expression in a concentration-dependent manner, suggesting its potential relevance to wrinkle-related skin protection. Although this observation supports a beneficial effect of SN in the UVB model, the precise molecular basis for MMP-1 suppression remains to be clarified. Proper moisturization is another key determinant of healthy skin. HAS-1 is involved in the synthesis of hyaluronic acid, a major component of the extracellular matrix that contributes to water retention, barrier support, and tissue repair [12, 13, 15]. Our results showed that SN increased HAS-1 expression in a concentration-dependent manner, indicating its possible moisturizing-related activity in keratinocytes. In addition, SN modulated NF-κB signaling under the tested conditions, which may be relevant to the regulation of HAS-1 expression. However, the present data do not establish a direct causal relationship between NF-κB activation and SN-induced HAS-1 upregulation.

Efficient wound repair is essential for maintaining skin integrity and function [19]. In the scratch assay, SN promoted wound closure in keratinocytes compared with the untreated group, indicating wound-healing–related activity *in vitro*. This finding suggests that SN may support keratinocyte migration or recovery under the experimental conditions used in this study, although the detailed mechanism underlying this response was not further investigated.

LC-Q-TOF-MS analysis tentatively identified several candidate constituents, including pectolinarin and aloeresin-related compounds. Although these compounds have been associated with skin-related bioactivities in previous studies, their direct contribution to the effects of SN remains to be determined.

SN also modulated selected inflammation- and sensory-related markers in keratinocyte-based assays. In particular, SN reduced IL-4 and IL-13 expression, whereas the responses of IL-31 and TRPV1 were less consistent across the tested concentrations. Therefore, the present data support modulation of selected IL-17A-responsive markers rather than a comprehensive anti-inflammatory mechanism. Further studies will be needed to clarify the biological significance of these responses.

The present study also showed that SN modulated NF-κB-, AP-1-, and CREB-related signaling responses, as well as MAPK-associated signaling molecules, under the tested conditions. However, these signaling changes should be interpreted with caution. In particular, the suppression of UVB-induced MMP-1 expression and the activation of AP-1/MAPK-related signaling were observed under different experimental contexts. Specifically, MMP-1 suppression was detected in UVB-irradiated HaCaT cells, whereas AP-1 reporter activation was measured in HEK293T cells and MAPK/AP-1-related phosphorylation events were examined in HaCaT cells under non-UVB conditions. Therefore, these findings are more appropriately interpreted as distinct context-dependent effects of SN rather than as evidence of a single direct signaling pathway linking AP-1 activation to MMP-1 suppression. Accordingly, the present data do not support a non-canonical model in which AP-1 activation directly mediates the suppression of MMP-1.

Taken together, the present study suggests that SN exerts multiple beneficial *in vitro* effects in keratinocyte-based assays, including UVB-protective, moisturizing-related, anti-wrinkle–related, and wound-healing–related activities. Nevertheless, this study has several limitations. The experiments were restricted to *in vitro* models, intracellular ROS levels were not directly measured, and the direct causal roles of individual signaling pathways were not established. In addition, detailed strain-level information for SN could not be disclosed because the formulation is proprietary, which limits full reproducibility and precise attribution of the observed biological effects to individual microbial strains or their derived components. Therefore, further studies will be required to clarify the precise mechanisms of SN and to validate its biological effects in more advanced experimental systems, including appropriate *in vivo* models.

## Conclusion

**Fig. 7:**
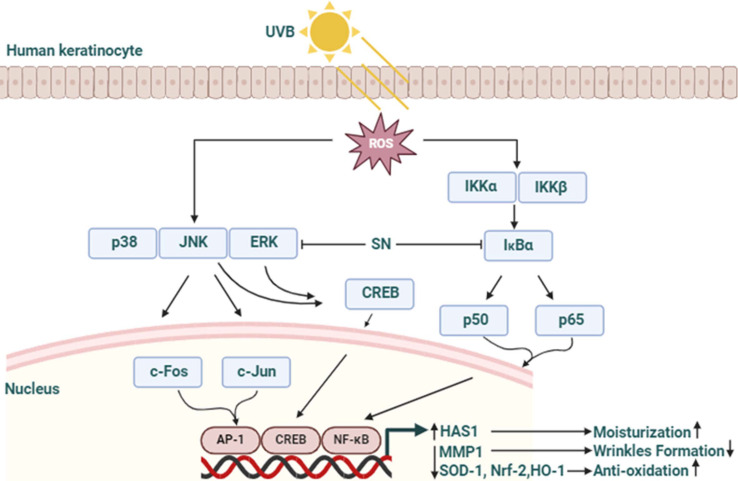
Summary of observed *in vitro* effects and associated signaling responses.

In conclusion, SN shows multiple beneficial *in vitro* effects in keratinocyte-based assays, including UVB-protective, moisturizing-related, anti-wrinkle–related, and wound-healing–related activities (Fig. 7). These findings suggest that SN may serve as a candidate fermentation-derived material for further skin-related investigation. However, additional studies are needed to clarify its precise mechanisms, validate its effects in more advanced experimental systems, and further assess its safety in skincare applications.

## Figures and Tables

**Table 1:** Primer sequences used in this study.

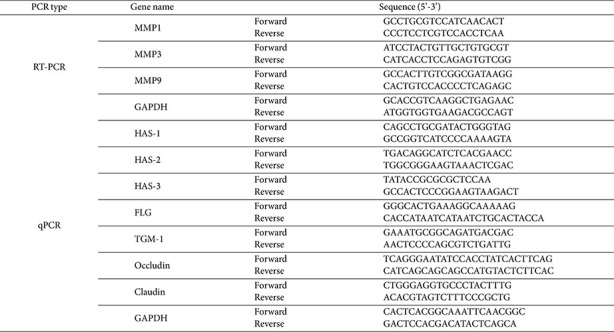

**Table 2:** LC-Q-TOF-MS analysis of SN components.